# Association between sleep duration and sarcopenic obesity: The mediating role of hemoglobin level

**DOI:** 10.1371/journal.pone.0347177

**Published:** 2026-04-27

**Authors:** Xiaojiang Zhao, Jun Chen, Lei Zhang, Hebao Wen, Laiguo Han, Hong Ding

**Affiliations:** 1 Department of Physical Education and Arts, Bengbu Medical University, Bengbu, China; 2 Innovation Center for Integration of Sports and Medicine, Capital University of Physical Education and Sports, Beijing, China; Covenant University, NIGERIA

## Abstract

**Background:**

Sarcopenic obesity (SO) is a major global health concern linked to sleep duration. This study examined the association between sleep duration and SO in middle-aged and older Chinese adults, with hemoglobin level as a mediating factor.

**Methods:**

This research examined data from the 2015 China Health and Retirement Longitudinal Study (CHARLS), focusing on individuals aged 45 years and older. We investigated the link between sleep duration, hemoglobin level, and SO using multivariate logistic regression, adjusting for confounders. A mediation model assessed hemoglobin’s role in the sleep duration-SO relationship. Nonlinear relationships were explored with restricted cubic spline fitting, and likelihood ratio tests evaluated threshold effects. Subgroup analysis examined group heterogeneity.

**Results:**

After adjusting for covariates, both sleep duration and hemoglobin level were negatively associated with SO. For each unit increase in sleep duration, the association with SO decreased by 9% (OR = 0.91, 95%CI: 0.87–0.95, p < 0.001). For each unit increase in hemoglobin level, the association was reduced by 25% (OR = 0.75, 95%CI: 0.71–0.80, p < 0.001). Hemoglobin had an indirect effect on the relationship between sleep duration and SO, accounting for 11.01% of the total effect variation (−1.20 × 10⁻³, 95%CI: −2.20 × 10⁻³ to 3.30 × 10⁻⁴).

**Conclusion:**

The study found that both sleep duration and hemoglobin level were negatively associated with SO, and hemoglobin level mediated the relationship between sleep duration and SO.

## Introduction

Sarcopenic obesity (SO) [1], a geriatric condition with clinical importance characterized by the simultaneous presence of decreased muscle mass (sarcopenia) and increased body fat (obesity), represents a significant threat to aging societies. Progressive adipose tissue accumulation coupled with muscle atrophy during aging exacerbates the pathophysiology of SO. This syndrome has emerged as a critical public health concern, correlating with functional decline [2], compromised well-being [3], elevated multimorbidity susceptibility [4,5], and higher all-cause mortality [6]. Epidemiological studies report SO prevalence rates of 2.75–20% among older adults, with variability attributable to heterogeneous diagnostic parameters [7]. The socioeconomic impact of SO is profound: In 2000 alone, U.S. healthcare expenditures related to SO reached $18.5 billion [8]. Given global demographic shifts toward aging populations, SO prevalence is projected to rise, underscoring the urgency of proactive screening and preventive measures. Prioritizing early identification and intervention strategies could mitigate disease burden and reduce strain on healthcare systems, offering a viable approach to addressing this dual metabolic and musculoskeletal disorder.

Adequate sleep duration is essential for maintaining physiological and psychological health in middle-aged and older populations. Epidemiological studies demonstrate a U-shaped relationship between sleep duration and obesity risk, with both insufficient (<6 hours) and prolonged (>8 hours) sleep durations correlating with elevated adiposity [9,10]. Furthermore, sleep patterns show significant associations with sarcopenia prevalence: individuals sleeping <6 hours nightly exhibit 2.76-fold higher odds of sarcopenia, while those exceeding 8 hours have 1.89-fold increased risk compared to normative sleepers (6–8 hours) [11,12]. Given sleep’s dual association with adiposity and muscle atrophy, circadian rest irregularities may serve as a modifiable factor in SO pathogenesis, warranting mechanistic investigations into their synergistic effects [9–12].

Sleep duration has increasingly been recognized as a crucial factor that affects various health-related parameters, including hemoglobin levels, especially within middle-aged and older adult populations. A study investigating the relationship between self-reported sleep duration and anemia in individuals aged 50 years and older in the UK found that shorter sleep duration was associated with lower hemoglobin levels [13]. Previous research has also indicated that longer sleep duration may elevate hemoglobin levels, potentially due to the restorative qualities of sleep [14]. The underlying mechanisms linking sleep duration and hemoglobin levels may involve inflammatory factors [14], lipid metabolism [15], and exercise and dietary habits [16].

Hemoglobin concentration is a well-established parameter utilized by clinical practitioners to diagnose anemia. Given the association between anemia and sarcopenia, it is plausible that reduced hemoglobin levels may correlate with sarcopenia [17]. Reduced hemoglobin concentrations impair oxygen transport to skeletal muscle tissue, adversely affecting muscular strength—a phenomenon documented in chronic hypoxic conditions [18]. Furthermore, anemia demonstrates positive correlation with elevated inflammatory biomarkers, potentially inducing detrimental effects on musculoskeletal integrity and functional capacity [18]. Additionally, research suggests that insulin resistance can affect hemoglobin levels, particularly in obese individuals, and is frequently associated with dysregulated blood glucose levels, potentially impacting hemoglobin glycation [19]. Sleep duration may influence hemoglobin homeostasis via multiple pathways—including systemic inflammation, endocrine rhythm, and lifestyle-related nutrient metabolism for hematopoiesis [20]. As the primary oxygen carrier, reduced hemoglobin can cause skeletal muscle hypoxia, impairing metabolic function and potentially triggering catabolism [21,22]. Moreover, chronic low-grade inflammation—common to both anemia and SO—may fuel a self-reinforcing vicious cycle. We therefore hypothesize that hemoglobin level mediates the association between sleep duration and SO.

This investigation analyzed data from the 2015 China Health and Retirement Longitudinal Study (CHARLS) to examine associations between sleep duration, hemoglobin level, and SO in adults aged ≥45 years. A mediation analytical framework was implemented to assess hemoglobin’s potential mediating role in the sleep duration-SO relationship.

## Study methodology

### Data collection

**Fig 1:**
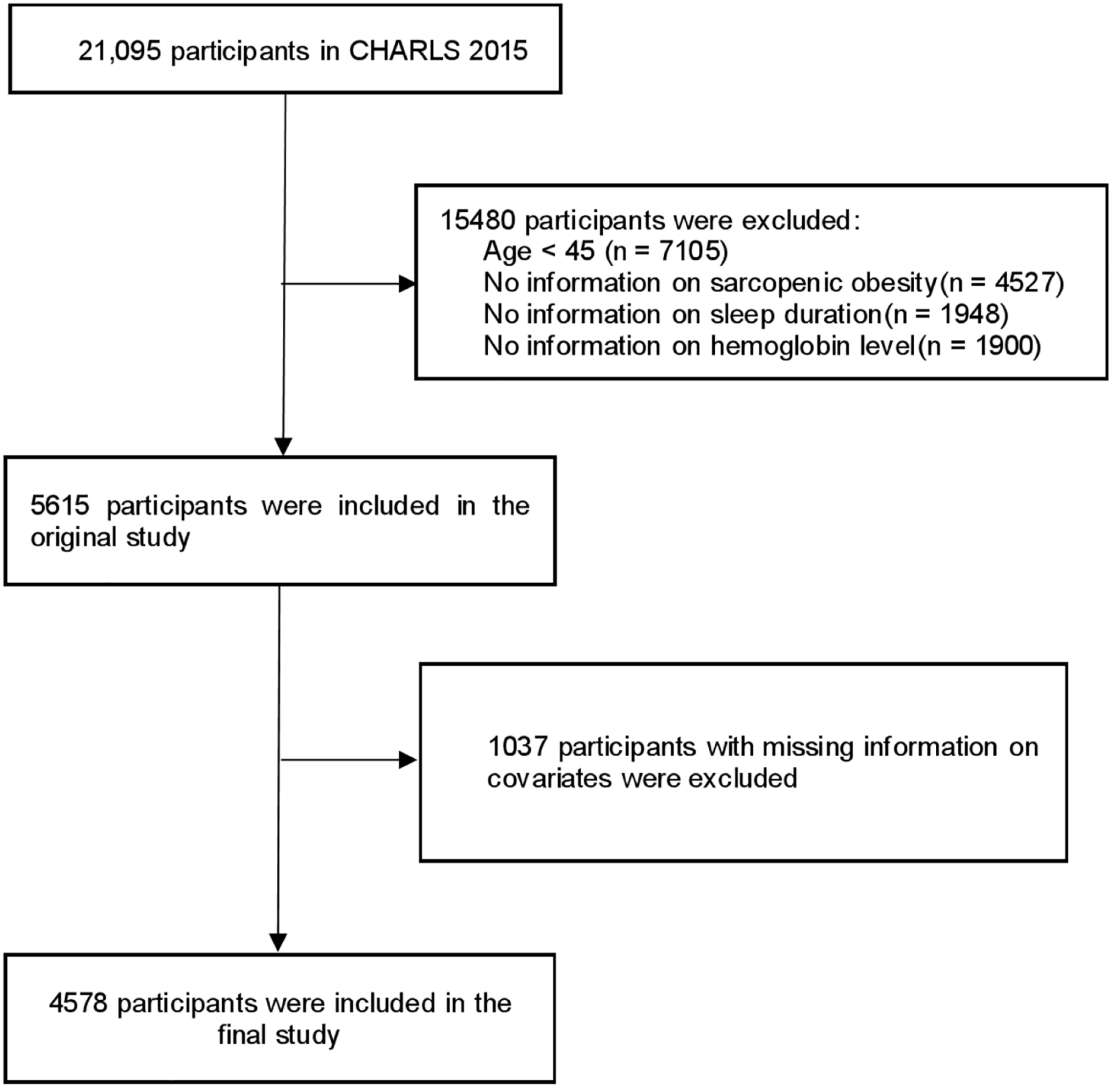
Flowchart of the participants selection process. Abbreviations: CHARLS, China health and retirement longitudinal study.

This analysis adopted a cross-sectional design, utilizing data obtained from the CHARLS. As a longitudinal survey that is nationally representative and focuses on Chinese adults aged 45 years and above, CHARLS aims to provide a detailed, publicly available micro-level dataset that is both accurate and reflective of the middle-aged and older population. A multistage probability-proportional-to-size (PPS) sampling strategy was employed across 28 provincial-level regions to ensure demographic diversity. Comprehensive methodological details regarding CHARLS have been extensively documented in previous research [23]. The CHARLS study received approval from the Peking University Ethics Committee (IRB00001052–11015), and all participants provided informed written consent. The inaugural national data collection occurred in 2015 with an initial cohort of 21,095 respondents. For analytical rigor, exclusion criteria were applied sequentially: individuals below 45 years (n = 7,105), those with incomplete SO assessments (n = 4,527), undocumented sleep duration (n = 1,948), unspecified hemoglobin level data (n = 1,900), and missing covariate information (n = 1,037) were omitted. This selection process yielded a final analytical cohort of 4,578 middle-aged and older participants, with the complete exclusion pathway illustrated in Fig 1.

### Assessments

#### Sarcopenia obesity.

Sarcopenia diagnosis followed the 2019 Asian Working Group for Sarcopenia (AWGS) guidelines [24], requiring either reduced appendicular skeletal muscle mass (ASM) or co-occurring deficits in handgrip strength (HGS) and gait speed (GS). ASM quantification utilized a population-specific equation validated for Chinese demographics:

ASM = 0.193 × Weight (kg)+0.107 × Height (cm)−4.157 × Sex (male = 1, female = 2)−0.037 × Age (years)−2.631 [25,26], with the appendicular muscle mass index (ASMI) calculated as ASM divided by height squared (m²) [27,28]. Sex-specific ASMI thresholds (men: < 7.0 kg/m²; women: < 5.34 kg/m²) were determined using the cohort’s lowest 20th percentile [29]. HGS was evaluated using the Yuejian™ WL-1000 dynamometer (Nantong Yuejian Co., China) [23]. Participants stood while performing two maximum-effort trials for each hand, and the highest value obtained from either hand was recorded. According to the AWGS 2019 criteria, low grip strength was defined as less than 28 kg for men and less than 18 kg for women [24]. GS was assessed as the average of two 2.5-meter walks performed at a normal pace, with slow walking speed categorized as less than 1.0 m/s [24]. Based on World Health Organization (WHO) standards for Asian populations, obesity was identified by a body mass index (BMI) of 25 kg/m² or higher [30]. BMI was computed by dividing an individual’s weight in kilograms by the square of their height in meters. In this study, participants were classified as having SO if they met the criteria for both sarcopenia and obesity.

#### Sleep duration.

Sleep duration data were derived from the CHARLS lifestyle and health module, specifically capturing nocturnal rest patterns through the query: “During the past month, how many hours of actual sleep did you get nightly (average per night)?” Responses, self-reported in hourly and minute increments, were stratified into three categories: short (＜6 hours/night), medium (6–8 hours/night), and long (＞8 hours/night) sleep durations [31]. This tripartite classification aligns with epidemiological standards for analyzing sleep-related health outcomes.

#### Hemoglobin level.

The CHARLS protocol, implemented in partnership with the Chinese Center for Disease Control and Prevention (China CDC), standardized venous blood sample collection and processing. Venous blood draws yielded three aliquots per participant: one aliquot underwent refrigerated storage at 4°C during transport to regional CDC facilities for immediate complete blood count analysis (median processing interval: 97 minutes), while the remaining two aliquots were cryopreserved at −80°C for subsequent biomarker analyses at a nationally certified laboratory affiliated with Capital Medical University. Cystatin C—a protein biomarker linked to muscle metabolism in chronic disease populations [32]—was quantified via particle-enhanced immunonephelometry (detection range: 0.5–8.0 mg/L). Hemoglobin levels were derived from complete blood count metrics using standardized hematological protocols. According to WHO standards, anemia is defined by hemoglobin levels below 12.0 g/dL for women and 13.0 g/dL for men [33]. Complete blood count analysis was performed at regional CDC laboratories using an automated hematology analyzer. The instrument is widely used in large-scale epidemiological studies and is regularly calibrated according to the manufacturer’s guidelines. The internal quality control data from the participating laboratories showed that the intra-assay coefficient of variation (CV) for hemoglobin measurements was less than 2% and the inter-assay CV was less than 3%. These values are within acceptable clinical and research ranges.

### Control variables

The baseline assessment captured sociodemographic and clinical variables, including age, physical activity (PA), gender, residential classification (urban/rural), and marital status (married/cohabiting versus unmarried/widowed/divorced). Health behavior covariates encompassed comorbidity burden (none, single, multiple), smoking status (current smoker/non-smoker), alcohol consumption frequency (non-drinker, <1/month, ≥ 1/month), and educational attainment (≤primary school or ≥middle school). Chronic disease prevalence was ascertained through self-reported physician diagnoses of fourteen non-communicable conditions spanning cardiometabolic, pulmonary, hepatic, renal, oncological, neurological, and musculoskeletal pathologies. Anthropometric measures (height, weight) obtained through standardized protocols facilitated BMI derivation (weight[kg]/height[m]²).

### Statistical methods

In this cross-sectional study, all statistical analyses were performed utilizing R version 4.3.3 and Free Statistics software version 2.0. The `ggplot2` package (version 3.5.0) was used for data visualization. Descriptive statistics for sample characteristics are presented as means with standard deviations and medians with interquartile ranges (IQR) for continuous variables and as frequencies and percentages for categorical variables. Spearman’s rank correlation coefficient was employed to evaluate the relationships between the primary variables, while multivariable logistic regression was used to examine the associations between sleep duration, hemoglobin level, and the outcome variable, SO. The magnitude of these associations was quantified using odds ratios (OR). Four asymptotic adjustment models were applied: the unadjusted model; Model 1, which included adjustments for age, sex, educational level, and marital status; Model 2, which further adjusted for smoking and drinking status; and Model 3, which additionally accounted for BMI and 14 chronic diseases. Multicollinearity was assessed via variance inflation factors (VIF), with values of >5 indicating collinearity. The selection of covariates for adjustment was based on a priori clinical knowledge and existing literature, to minimize confounding bias [28,34]. Restricted cubic splines (RCS) were used to model potential nonlinear associations between sleep duration and SO, and between hemoglobin levels and SO, with the “rms” package and adjustment variables from Model 3. There were 2 nodes for sleep duration and 2 nodes for hemoglobin level. Likelihood ratio tests (LRT) were conducted to evaluate the threshold effects of sleep duration and hemoglobin level on SO. Stratified subgroup analyses were conducted to examine effect heterogeneity across predefined demographic and health-related categories. We applied the Bonferroni correction, with the calculation method being 0.05 divided by the number of sub-samples [35]. In this case, as there were 10 subgroups, the corrected value was 0.05/10. Therefore, only variables with a p < 0.005 were considered. Subsequently, a Baron and Kenny mediation model was employed to investigate the threshold effects of hemoglobin levels on sleep duration and SO [36]. Specifically, the mediating role of hemoglobin levels in the association between sleep duration and SO was analyzed. The mediation package of R software performed the mediation analysis. The mediator effect was quantified by calculating the proportion of variance explained by the mediator, and its significance was evaluated using bootstrap resampling (1000 replications). p < 0.05 was considered statistically significant. We further compared the characteristics of excluded and included participants across demographics, lifestyle habits, clinical factors, and key variables to assess potential selection bias.

## Result

### Characteristics of the study participants

The cohort was stratified into non-SO and SO subgroups, with demographic and clinical profiles summarized in Table 1. The analysis included 4,579 adults (mean age 61.6 ± 9.8 years), comprising 3,599 non-SO individuals (mean age 60.0 ± 9.3 years) and 979 classified as SO (mean age 67.3 ± 9.5 years). Gender distribution differed markedly: males constituted 50.7% (n = 1,825) of the non-SO group versus 42.4% (n = 415) in the SO group, while females represented 49.3% (n = 1,774) and 57.6% (n = 564), respectively. A majority resided in rural regions (62.8%) and were married and cohabiting with spouses (85.5%). The two groups differed significantly in age, sex, marital status, education, smoking, alcohol use, BMI, PA and chronic disease prevalence (p < 0.001). Notably, the average sleep duration was significantly different between the non-SO group (6.6 ± 1.7 hours) and the SO group (6.2 ± 2.2 hours), with p < 0.001. Likewise, hemoglobin levels differed significantly, averaging 14.6 ± 1.6 g/dl in the non-SO group and 13.6 ± 1.9 g/dl in the SO group (p < 0.001). Sensitivity analysis revealed no significant multicollinearity (the maximum VIF = 1.388 < 5), supporting the robustness of the multivariate estimates (S1 Table). The baseline characteristics of included and excluded participants are shown in S2 Table.

**Table 1:** Characteristics of study participants according to sarcopenic obesity.

Variables	Total	Non-SO	SO	P value
n = 4,578	n = 3599	n = 979
Age, Mean ± SD	61.6 ± 9.8	60.0 ± 9.3	67.3 ± 9.5	< 0.001
Sex, n (%)				< 0.001
Female	2344 (51.2)	1774(49.3)	564 (57.6)	
Male	2234 (48.8)	1825 (50.7)	415 (42.4)	
Residence, n (%)				0.141
Rural	2877 (62.8)	2242 (62.3)	635 (64.9)	
Urban	1701 (37.2)	1357 (37.7)	344 (35.1)	
Marital status, n (%)				< 0.001
Married and living with a spouse	3916 (85.5)	3197 (88.8)	719 (73.4)	
Married but living without a spouse	135 (2.9)	109 (3)	26 (2.7)	
Single, divorced, and windowed	527 (11.5)	293 (8.1)	234 (23.9)	
Education Status, n (%)				< 0.001
Elementary school or below	2823 (61.7)	2032 (56.5)	791 (80.8)	
Middle school or above	1755 (38.3)	1567 (43.5)	188 (19.2)	
Smoking Status, n (%)				< 0.001
Non-smoker	1212 (26.5)	640 (17.8)	572 (58.4)	
Smoker	3366 (73.5)	2959 (82.2)	407 (41.6)	
Drinking Status, n (%)				< 0.001
drinker	2451 (53.5)	2152 (59.8)	299 (30.5)	
Non-drinker	2127 (46.5)	1447 (40.2)	680 (69.5)	
BMI(kg/m^2^), Median (IQR)	23.5 (21.2, 26.1)	23.2 (20.9, 25.6)	25.2 (22.6, 28.4)	< 0.001
PAL				< 0.001
Low PAL	592 (12.9)	458 (12.7)	121 (12.4)	
Moderate PAL	1442 (31.5)	1094 (30.4)	348 (35.5)	
High PAL	2544 (55.6)	2047 (56.9)	510 (52.1)	
Number of chronic conditions, n (%)				< 0.001
0	1323 (28.9)	1129 (31.4)	194 (19.8)	
1	1123 (24.5)	901 (25)	222 (22.7)	
≥2	2132 (46.6)	1569 (43.6)	563 (57.5)	
Sleep duration(hrs), Mean ± SD	6.5 ± 1.8	6.6 ± 1.7	6.2 ± 2.2	< 0.001
Hemoglobin level(g/dl), Mean ± SD	14.4 ± 1.7	14.6 ± 1.6	13.6 ± 1.9	< 0.001

Abbreviations: SO, sarcopenic obesity; BMI, body mass index; hrs, hours; PAL, physical activity level.

### The relationship between important variables

Table 2 shows the association between sleep duration, hemoglobin level, and SO. The study assessed relationships between nocturnal sleep duration and SO in 4,578 participants. Unadjusted models demonstrated a significant inverse correlation (OR = 0.89, 95%CI: 0.85–0.92, *P* < 0.001). Initial covariate adjustment (Model 1: age, sex, education, marital status) yielded a modest attenuation of this association (OR = 0.92, 95%CI: 0.88–0.95, *P* < 0.001). Progressive adjustments for lifestyle factors (Model 2: smoking, alcohol use) and clinical variables (Model 3: BMI, chronic diseases) produced comparable results, with odds ratios of 0.93 (95%CI: 0.89–0.97) and 0.91 (95%CI: 0.87–0.95), respectively, both retaining statistical significance (*P* < 0.001). This stability across models suggests that covariate inclusion minimally influenced the observed inverse sleep duration-SO relationship.

**Table 2:** Associations of sleep duration and hemoglobin level with sarcopenic obesity.

Variables	No	Unadjusted		Model 1		Model 2		Model 3	
OR(95% CI)	p value	OR (95% CI)	p value	OR(95% CI)	p value	OR (95% CI)	p value
Sleep duration	4578	0.89(0.85 ~ 0.92)	<0.001	0.92(0.88 ~ 0.95)	<0.001	0.93(0.89 ~ 0.97)	<0.001	0.91 (0.87 ~ 0.95)	<0.001
Hemoglobin level		0.69 (0.66 ~ 0.72)	<0.001	0.78(0.74 ~ 0.82)	<0.001	0.85(0.82 ~ 0.91)	<0.001	0.75 (0.71 ~ 0.80)	<0.001

Model 1: adjusted for age, gender, educational level, marital status, and residence. Model 2: adjusted for model 1 + smoking status and drinking status. Model 3: adjusted for model 2 + BMI, physical activity level, and 14 chronic diseases. Abbreviations: OR, odds ratio; 95% CI, 95% confidence interval; BMI, body mass index.

Hemoglobin level demonstrated an inverse relationship with SO risk (OR = 0.69, 95%CI: 0.66–0.72, P < 0.001). Progressive adjustments revealed attenuated yet persistent associations: Model 1 (adjusted for demographic variables) yielded OR = 0.78 (95%CI: 0.74–0.82; P < 0.001), Model 2 (additional lifestyle adjustments) showed OR = 0.85 (95%CI: 0.82–0.91; P < 0.001), and Model 3 (full clinical adjustments) maintained significance at OR = 0.75 (95%CI: 0.71–0.80; P < 0.001). This persistent inverse association underscores hemoglobin’s robust, independent linkage to SO risk despite sequential covariate adjustments.

**Fig 2:**
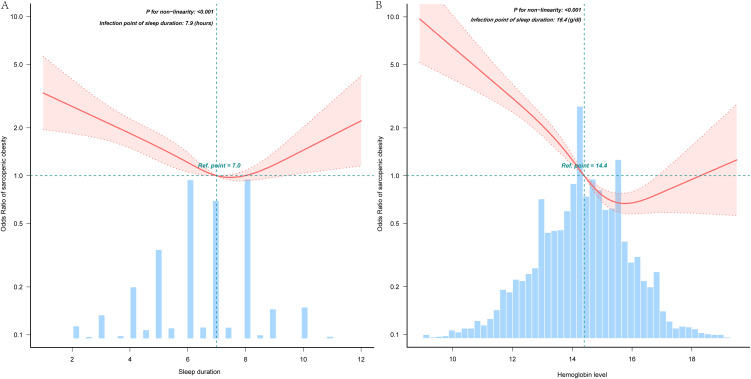
Nonlinear associations of sleep duration and sarcopenic obesity (A), nonlinear associations of hemoglobin level and sarcopenic obesity (B). The median was used as the reference point. Solid and dashed lines represent the predicted value and 99% CI, respectively. Orange bars represent the distribution of the entire cohort adjusted for age, gender, educational level, marital status, residence, smoking status, drinking status, BMI, and 14 chronic diseases, only 99% of the data is displayed.

Analyses employing RCS identified a nonlinear dose-response relationship between sleep duration and SO, with threshold effects achieving statistical significance (*P* < 0.001; Fig 2A). Similarly, hemoglobin level demonstrated a nonlinear association with SO risk, exhibiting a curvilinear pattern (*P* < 0.001; Fig 2B).

Threshold effect analysis, detailed in Table 3, identified an inverse association between sleep durations below 7.9 hours and SO (OR = 0.81, 95%CI: 0.75–0.87; P < 0.001), indicating lower odds of SO per incremental hour of sleep within this range. Conversely, sleep durations exceeding 7.9 hours exhibited a positive association with SO (OR = 1.19, 95%CI: 1.01–1.41; P = 0.04), suggesting higher odds of SO for each additional hour of prolonged sleep.

**Table 3:** Threshold effect analysis of the relationship of sleep duration with sarcopenic obesity.

Sleep duration	Adjusted Model	
OR (95% CI)	P value
< 7.9	0.81(0.75 ~ 0.87)	<0.001
≧7.9	1.19(1.01 ~ 1.41)	0.04
Likelihood Ratio test		<0.001

Adjusted for age, gender, educational level, marital status, residence, smoking status, drinking status, BMI, physical activity level, and 14 chronic diseases, Only 99% of the data is displayed. Abbreviations: OR, odds ratio; 95% CI, 95% confidence interval.

Table 4 presents the relationship between hemoglobin levels below 16.4 g/dL and SO in the threshold analysis (OR = 0.67, 95%CI: 0.62–0.72, p < 0.001). This indicates substantially lower odds of SO for each unit increase in hemoglobin level within this range. In contrast, for participants with hemoglobin levels of 16.4 g/dL or higher, no statistically significant association with SO was observed (OR = 1.03, 95%CI: 0.64–1.65, p = 0.90).

**Table 4:** Threshold effect analysis of the relationship of hemoglobin level with sarcopenic obesity.

Hemoglobin level	Adjusted Model	
OR (95% CI)	P value
< 16.4	0.67(0.62 ~ 0.72)	<0.001
≧16.4	1.03(0.64 ~ 1.65)	0.90
Likelihood Ratio test		0.05

Adjusted for age, gender, educational level, marital status, residence, smoking status, drinking status, BMI, physical activity level, and 14 chronic diseases, Only 99% of the data is displayed. Abbreviations: OR, odds ratio; 95% CI, 95% confidence interval.

S3 Table presents the subgroup analysis of the association between sleep duration and SO, showing homogeneity among groups (interaction P > 0.005). In addition, S4 Table elucidates the findings of subgroup analyses exploring the relationship between hemoglobin levels and SO, highlighting that educational status (P = 0.001 for interaction), smoking (P < 0.001 for interaction), alcohol consumption (P < 0.001 for interaction), and BMI (P < 0.001 for interaction) significantly modulated the association between hemoglobin levels and SO risk. S5 Table shows the coefficients and standard errors of all covariates.

### The relationship between sleep duration and sarcopenic obesity was mediated by hemoglobin level

Table 5 delineates the interrelationships between baseline sleep duration, hemoglobin level, and SO. Sleep duration demonstrated a significant inverse correlation with SO (r = −0.09, P < 0.001). Concurrently, longer sleep durations were positively associated with hemoglobin levels (r = 0.07, P < 0.001), while hemoglobin level exhibited an inverse relationship with SO risk (r = −0.24, P < 0.001). These correlational patterns highlight hemoglobin’s potential role as a mediator in the sleep duration-SO pathway.

**Table 5:** Associations among sleep duration and hemoglobin level with sarcopenic obesity.

Variables	Sleep duration	Hemoglobin level	Sarcopenic obesity
Sleep duration	1.00		
Hemoglobin level	0.07***	1.00	
Sarcopenic obesity	−0.09***	−0.24***	1.00

*** P-value < 0.001.

**Fig 3:**
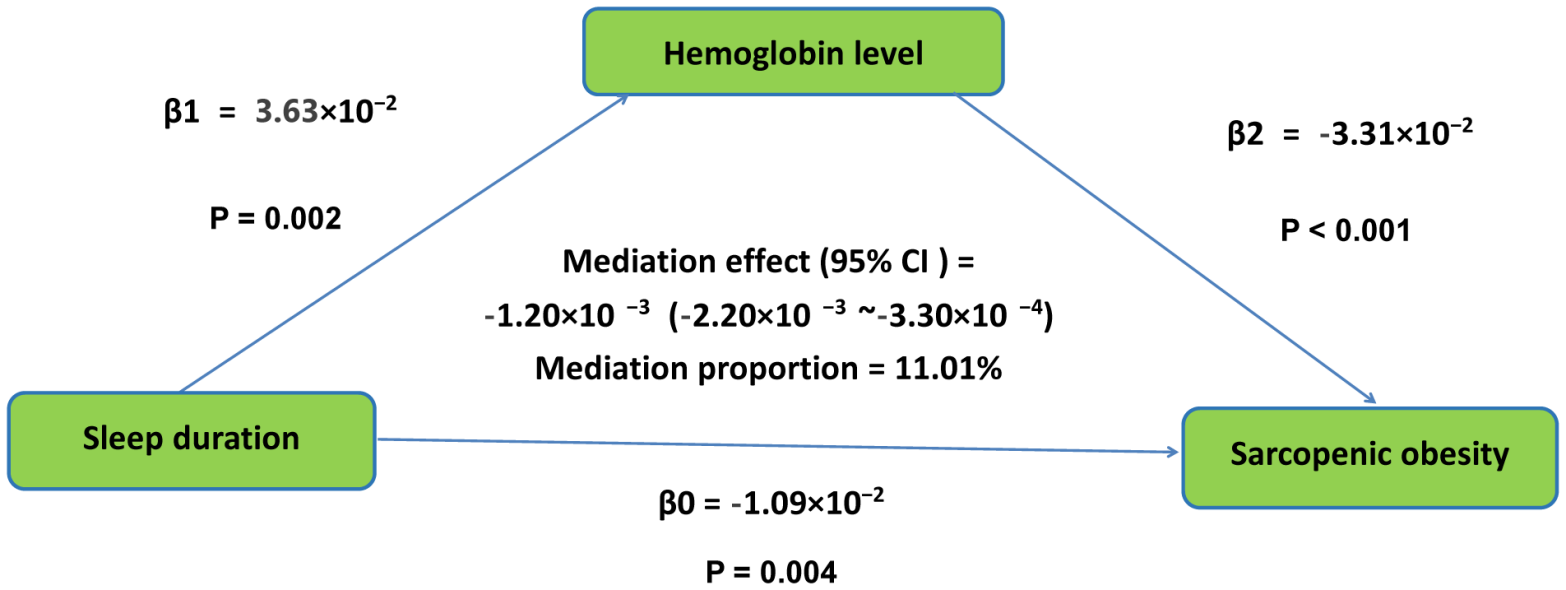
The conceptual framework of the mediation models. β0 was the total effect of sleep duration on sarcopenic obesity; β1 represents the effect of sleep duration on hemoglobin level; β2 represents the effect of hemoglobin level on sarcopenic obesity. The mediation effect was computed as the product of “β1” and “β2”(β1 × β2), and the mediation proportion was calculated as the ratio of the mediation effect product to total effects [(β1 × β2)/β0].

Bootstrap resampling demonstrated the aggregate effect of sleep duration on SO with β = −1.09 × 10⁻² (P = 0.004). Hemoglobin level mediated this relationship, exhibiting a partial mediation effect of β = −1.20 × 10⁻³ (95%CI: 95% CI = −2.20 × 10⁻³ to −3.30 × 10⁻⁴), explaining 11.01% of the total variance. The standardized total effect of sleep duration on SO was −0.0198 (per SD increase), with a standardized indirect effect via hemoglobin of −0.0022, corresponding to a 0.22% lower SO per SD increase in sleep duration mediated by hemoglobin. For each additional hour of sleep, the mediated reduction in SO was approximately 0.12%, based on a sleep duration SD of 1.8 hours. This intermediary pathway—linking sleep duration to SO through hemoglobin—is graphically represented in Fig 3. In addition, S1 Fig shows the mediation analysis results by anemia status.

## Discussion

This research represents the first investigation into the observed relationships among sleep duration, hemoglobin level, and SO within an aging Chinese population, utilizing CHARLS data from 2015. The findings indicate a significant cross-sectional association between sleep duration and the risk of SO. Furthermore, the exploratory analysis suggested that hemoglobin level may statistically account for part of the relationship between sleep duration and SO, which is consistent with the initial hypothesis.

The multivariate logistic regression analysis revealed a negative cross-sectional association between sleep duration and the prevalence of SO in individuals aged 45 years and older (OR = 0.91, 95%CI: 0.87–0.95, P < 0.001). The RCS model combined with LRT revealed a nonlinear dose-response dynamic relationship, with a critical inflection point at 7.9 hours/night. Specifically, longer sleep duration (≥7.9 hours) was associated with higher odds of SO, while shorter sleep duration (<7.9 hours) was associated with lower odds. The current literature on the relationship between sleep duration and SO is still sparse. Earlier study in adults aged ≥60 years found that sleep duration (≥9 hours) was positively associated with SO risk, while sleep duration below this threshold was not significantly associated, a difference that may be attributed to cohort age differences and covariate adjustment methods [37]. Additional evidence from meta-analyses supporting the U-shaped relationship between sleep duration and sarcopenia may help explain the nonlinear pattern observed in the current study [38].

The biological pathways connecting sleep duration to sarcopenic obesity (SO) pathogenesis remain partially understood, though evidence suggests shared mechanisms between sarcopenia and obesity may collectively contribute to SO progression. Prolonged sleep durations associated with insulin resistance and chronic low-grade inflammation [39,40], both implicated in muscle loss and adiposity. Insulin resistance inversely associates with circulating insulin-like growth factor 1 (IGF-1) levels [41], a pivotal regulator of muscle anabolism through the IGF-1/phosphatidylinositol 3-kinase (PI3K)/protein kinase B (Akt)/mammalian target of rapamycin (mTOR) signaling axis. This metabolic disruption concurrently suppresses anabolic signaling while activating fork head box O (FOXO)-mediated transcription of proteolytic factors like atrogin-1 and Muscle RING-Finger 1 (MuRF1), accelerating muscle catabolism [42]. Concurrently, elevated proinflammatory markers such as IL-6 exacerbate muscle degradation via ubiquitin-proteasome activation and nuclear factor-kappa B (NF-κB) pathway stimulation [43]. Sleep-related disturbances in testosterone synthesis may further upregulate myostatin and regulated in development and DNA damage responses 1 (REDD1), key drivers of proteolytic activity [42]. These intersecting pathways could potentially link sleep patterns to muscle and adipose tissue homeostasis, providing a plausible biological framework for the observed sleep-SO association.

On the other hand, evidence suggests an association between sleep duration and PA. A decline in PA is widely acknowledged as a significant factor contributing to the onset of sarcopenia and obesity [44,45]. Research has indicated that longer self-reported sleep durations are linked to lower levels of daily PA, with each extra hour of sleep corresponding to an average decrease of 29 minutes in PA [46–48]. As a result, PA could act as an intermediary in the association between excessive sleep and SO. The presence of excess body fat and reduced muscle mass/function, often stemming from insufficient PA, may interfere with regular sleep patterns via processes such as systemic inflammation, insulin resistance, and the buildup of neurotoxic metabolites that are not properly eliminated [49], thus forming a negative feedback loop. Furthermore, molecular clocks and circadian rhythms play a crucial role in preserving and modulating skeletal muscle function, as well as in governing lipid metabolism [50]. PA has the ability to modify the molecular clock in skeletal muscle, which subsequently influences the regulation of the circadian rhythm [51]. It has been suggested that exercise serves as a valuable approach to tackle sarcopenia and metabolic conditions by recalibrating the circadian clock [52,53]. Adequate sleep reflects stable circadian rhythm function. This rhythm is driven by the core biological clock and synchronized by environmental zeitgebers—especially daylight. Daytime light exposure, particularly outdoors, consolidates sleep structure, improves sleep efficiency, and maintains appropriate duration; insufficient exposure or circadian disruption directly impairs sleep [54]. PA serves a dual role: it acts as a behavioral zeitgeber to stabilize rhythms and is a primary source of daytime light exposure. Thus, declining PA in middle-aged and older adults may indirectly impair sleep via reduced light exposure and weakened circadian alignment. In turn, sleep loss or rhythm disruption exacerbates inflammation, insulin resistance, and metabolic dysfunction—fueling a vicious cycle with low hemoglobin and SO.

The link between sleep duration and hemoglobin levels involves complex physiological processes. Sleep affects systemic inflammation and immune function by regulating cytokines, which are crucial for inflammation. Short sleep can raise pro-inflammatory cytokines, potentially impacting hemoglobin levels by disrupting erythropoiesis, the production of red blood cells. Thus, insufficient sleep might contribute to anemia by hindering red blood cell production [55]. Sleep can also affect metabolic processes, including iron metabolism, which is vital for hemoglobin. Sleep deprivation may alter hormone levels like cortisol, impacting iron metabolism and hemoglobin levels. Moreover, sleep duration influences adipokines, hormones from fat tissue that affect inflammation and insulin resistance. Changes in adipokines like leptin and visfatin, linked to sleep duration, may indirectly impact hemoglobin by affecting iron metabolism and red blood cell production [56]. Furthermore, sleep duration affects hemoglobin levels through lifestyle factors like diet and exercise. Poor sleep can lead to unhealthy eating and less PA, impacting iron intake and hemoglobin [57].

Our mediation analysis, which should be interpreted as exploring a hypothesized pathway rather than proving causality, suggested that hemoglobin level partially accounted for the statistical association between sleep duration and SO. The indirect effect was small but statistically significant, with hemoglobin mediating approximately 11.01% of the total association. In standardized terms, each SD increase in sleep duration was associated with a 0.22% reduction in SO risk mediated through hemoglobin. This suggests that while hemoglobin plays a measurable mediating role. After comprehensive adjustment of covariates, for every 1 unit increase in hemoglobin level, the risk of SO decreased by 25% (OR = 0.75, 95%CI: 0.71–0.80; p < 0.001). We hypothesize that inflammation might be a common factor underlying both low hemoglobin levels and SO, given that both conditions involve elevated inflammatory markers. Both conditions involve inflammation, which can cause anemia of chronic disease with low hemoglobin. Elevated inflammatory markers like C-reactive protein and IL-6 in SO suggest chronic inflammation might lower hemoglobin levels [58,59]. Insulin resistance, common in SO, may hinder red blood cell production, resulting in lower hemoglobin level. The triglyceride-glucose index, a marker of insulin resistance linked to SO, suggests that insulin resistance may influence the connection between hemoglobin level and SO [28,60]. Moreover, hormonal changes, especially involving leptin, may affect the link between hemoglobin level and SO. Leptin, produced by fat tissue, is usually high in obesity and plays a role in red blood cell production. Elevated leptin may hinder red blood cell production, leading to lower hemoglobin level in those with SO [61,62].

To address public health challenges in middle-aged and older populations, holistic strategies should prioritize optimizing sleep duration through community-based education on sleep hygiene and circadian rhythm regulation, while avoiding extremes of insufficient or excessive sleep. Concurrently, hemoglobin level can be enhanced via anemia screening and dietary interventions emphasizing iron, folate, and vitamin B12 sources (e.g., lean meats, leafy greens), alongside light aerobic exercise to improve circulation. For SO prevention, multifaceted interventions are critical: combining high-quality protein intake (e.g., legumes, fish) with caloric control to balance nutrition, integrating resistance and aerobic training to maintain muscle mass and metabolic function, and systematically managing chronic diseases (e.g., monitoring BMI, blood glucose). Community-driven initiatives, including health registries, screenings, and educational workshops, should empower individuals to adopt preventive behaviors and ensure early intervention. These integrated approaches synergistically mitigate age-related health risks by addressing lifestyle, nutritional, and healthcare access disparities.

This study presents several benefits. Firstly, it uses longitudinal, population-based cohort data from China, boosting the results’ generalizability and empirical strength. Secondly, it is the first to systematically explore the links between sleep duration, hemoglobin levels, and SO risk in middle-aged and older adults. Lastly, it assesses hemoglobin level as a potential mediator, reinforcing the mechanistic understanding and offering a solid basis for SO prevention and improvement. Concurrently, this study acknowledges several limitations. Firstly, the research’s emphasis on middle-aged and older populations within China’s sociocultural and demographic context limits generalizability of findings to younger cohorts or diverse ethnic/cultural groups. Secondly, the cross-sectional survey design limits causal inference as it measures all variables simultaneously, preventing the establishment of time series and mechanistic pathways between exposures (e.g., sleep duration, hemoglobin level) and SO outcomes. Thirdly, unmeasured confounding factors—such as chronic inflammation, diet, and recent hospitalization—may bias the observed association and influence the mediation effect. Hemoglobin levels may be a consequence rather than a cause: reduced PA can diminish hematopoietic stimulation, which is linked to malnutrition and impaired vitamin B12 absorption. Moreover, light exposure is a key zeitgeber for regulating circadian rhythms, sleep homeostasis, and metabolic health. Although the model includes BMI and chronic diseases as proxies and CHARLS provides basic PA data, it lacks measurements of light exposure and objective circadian markers. Furthermore, data collection spanned multiple seasons, and seasonal variations may affect these behaviors. If these factors are associated with both sleep duration and SO, they could introduce residual confounding and bias the association estimates. Fourthly, included and excluded participants differed substantially in age, sex, marital status, education level, residence, and number of chronic conditions, which may introduce selection bias. Fifthly, this study focused on sleep duration and did not assess sleep phases—such as timing and differences between workdays and free days, known as “social jet lag”—which may be more important for metabolic health than duration alone. It also did not distinguish between participants’ active and retired status; since work obligations affect sleep regularity and opportunities, this lack of information may confound estimates of the association between sleep duration and SO, limiting a deeper understanding of the sleep-health relationship. Sixthly, hemoglobin measurements may be subject to circadian rhythm bias. Hemoglobin levels fluctuate physiologically over the day. Since the CHARLS database did not standardize blood collection times or record participants’ sleep chronotypes, we cannot exclude random errors from varying measurement times or differences in circadian phases—potentially biasing the observed associations. Lastly, sleep duration data were self-reported, which may introduce recall bias, and the absence of sleep quality measures could impact outcomes and mediation. The single average sleep metric used does not differentiate between workday and free-day patterns, potentially weakening observed associations or misclassifying long sleepers, particularly in this mixed employed-retired cohort.

Future studies should prioritize longitudinal designs to establish causality between sleep duration, hemoglobin level, and SO. Mechanistic investigations using biomarkers (e.g., inflammatory cytokines, insulin resistance markers) and omics approaches (genomics, proteomics) are needed to elucidate biological pathways. Randomized trials testing sleep optimization and anemia interventions could clarify their therapeutic potential. Expanding cohorts to diverse ethnicities and validating findings with objective measures (e.g., actigraphy, hemoglobin assays) would enhance generalizability. Integrating lifestyle factors (diet, PA) and genetic predispositions into models may refine risk stratification. Multidisciplinary collaborations will advance translational strategies for SO prevention. In addition, longitudinal studies should assess sleep duration separately for working and rest days and include measures of sleep regularity to clarify the causal relationship between sleep patterns and SO.

## Conclusion

This cross-sectional study found significant inverse associations between sleep duration and SO, and between hemoglobin level and SO, in a middle-aged and older Chinese population. Exploratory mediation analysis suggested that hemoglobin level partially accounted for the statistical association between sleep duration and SO. However, due to the study’s cross-sectional design, causal inferences cannot be made. The findings highlight the importance of considering sleep patterns and hemoglobin levels in the multifaceted assessment of SO risk. Future longitudinal and interventional studies are needed to determine whether optimizing sleep duration and maintaining adequate hemoglobin levels could play a role in SO prevention and management.

## Supporting information

S1 Table Collinearity analysis result. (DOC)

S2 Table Characteristics of study participants according to enrollees or non-enrollees. (DOC)

S3 Table Subgroup analysis of the association between sleep duration with and sarcopenic obesity. (DOC)

S4 Table Subgroup analysis of the association between hemoglobin level with and sarcopenic obesity. (DOC)

S5 Table Associations of sleep duration and hemoglobin level with SO. (DOC)

S1 Fig Mediation analysis stratified by anemia. (TIFF)

S1 Data Raw data. (CSV)
