# Supplementary material for: Association between sleep duration and sarcopenic obesity: The mediating role of hemoglobin level
Source: PLoS One. 2026 Apr 27;21(4):e0347177. doi: 10.1371/journal.pone.0347177 (PMC13119890; doi:10.1371/journal.pone.0347177)
Supplement: S1 Table — (DOC) [file pone.0347177.s001.doc]

**S1 Table. Collinearity analysis result**

| Term1 | VIF | DF | VIF^(1/(2*Df)) | Colinearity |
| --- | --- | --- | --- | --- |
| Crude | 1.02 | 1 | 1.01 | 0 |
| Age | 1.388 | 1 | 1.178 | 0 |
| Sex | 1 | 1 | 1 | 0 |
| Residence | 1.066 | 1 | 1.033 | 0 |
| Marital status | 1.104 | 2 | 1.025 | 0 |
| Education Status | 1.083 | 1 | 1.041 | 0 |
| Smoking Status | 1.014 | 1 | 1.007 | 0 |
| Drinking Status | 1.052 | 1 | 1.026 | 0 |
| BMI | 1.322 | 1 | 1.15 | 0 |
| PAL | 1.205 | 2 | 1.103 | 0 |
| Number of chronic conditions | 1.094 | 2 | 1.023 | 0 |
| Abbreviations: BMI, body mass index; PAL, physical activity level; VIF, variance inflation factors; df ,  degrees of freedom. | | | | |
