# Supplementary material for: Association between sleep duration and sarcopenic obesity: The mediating role of hemoglobin level
Source: PLoS One. 2026 Apr 27;21(4):e0347177. doi: 10.1371/journal.pone.0347177 (PMC13119890; doi:10.1371/journal.pone.0347177)
Supplement: S2 Table — (DOC) [file pone.0347177.s002.doc]

 S2 Table. Characteristics of study participants according to enrollees or non-enrollees

| Variables | Total | Included participants | Excluded participants | p |
| --- | --- | --- | --- | --- |
|  | n = 21095 | n = 4578 | n = 16517 |  |
| Age, Mean ± SD | 59.1 ± 10.8 | 61.6 ± 9.8 | 58.4 ± 10.9 | < 0.001 |
| Sex, n (%) |  |  |  | < 0.001 |
| Female | 11021 (52.3) | 547 (11.9) | 10474 (63.5) |  |
| Male | 10054 (47.7) | 4031 (88.1) | 6023 (36.5) |  |
| Residence, n (%) |  |  |  | < 0.001 |
| Rural | 12533 (59.5) | 2877 (62.8) | 9656 (58.5) |  |
| Urban | 8547 (40.5) | 1701 (37.2) | 6846 (41.5) |  |
| Marital status, n (%) |  |  |  | < 0.001 |
| Married and living with a spouse | 16974 (80.8) | 3916 (85.5) | 13058 (79.4) |  |
| Married but living without a spouse | 1304 ( 6.2) | 135 (2.9) | 1169 (7.1) |  |
| Single, divorced, and windowed | 2741 (13.0) | 527 (11.5) | 2214 (13.5) |  |
| Education Status, n (%) |  |  |  | < 0.001 |
| Elementary school or below | 14802 (70.3) | 2823 (61.7) | 11979 (72.7) |  |
| Middle school or above | 6258 (29.7) | 1755 (38.3) | 4503 (27.3) |  |
| Smoking Status, n (%) |  |  |  | < 0.001 |
| Non-smoker | 11888 (56.8) | 1212 (26.5) | 10676 (65.3) |  |
| Smoker | 9029 (43.2) | 3366 (73.5) | 5663 (34.7) |  |
| Drinking Status, n (%) |  |  |  | < 0.001 |
| drinker | 7355 (35.3) | 5421 (53.5) | 4904 (30.2) |  |
| Non-drinker | 13477 (64.7) | 2127 (46.5) | 11350 (69.8) |  |
| BMI(kg/m2), Median (IQR) | 23.6 (21.2, 26.2) | 23.5 (21.2, 26.1) | 23.6 (21.3, 26.3) | 0.372 |
| Number of chronic conditions, n (%) |  |  |  | < 0.001 |
| 0 | 7099 (33.7) | 1323 (28.9) | 5776 (35) |  |
| 1 | 4625 (21.9) | 1123 (24.5) | 3502 (21.2) |  |
| ≥2 | 9356 (44.4) | 2132 (46.6) | 7224 (43.8) |  |
| Sleep duration(hrs), Mean ± SD | 6.4 ± 1.9 | 6.5 ± 1.8 | 6.4 ± 2.0 | < 0.001 |
| Hemoglobin level(g/dl), Mean ± SD | 13.7 ± 1.9 | 14.4 ± 1.7 | 13.3 ± 2.0 | < 0.001 |
| Abbreviations: BMI, body mass index; hrs, hours. | | | | |
