# Supplementary material for: Association between sleep duration and sarcopenic obesity: The mediating role of hemoglobin level
Source: PLoS One. 2026 Apr 27;21(4):e0347177. doi: 10.1371/journal.pone.0347177 (PMC13119890; doi:10.1371/journal.pone.0347177)
Supplement: S3 Table — (DOC) [file pone.0347177.s003.doc]

S3 Table. Subgroup analysis of the association between sleep duration with and sarcopenic obesity

| Subgroup | n.total | n.event | OR (95%CI) | P for interaction |
| --- | --- | --- | --- | --- |
| Age |  |  |  | 0.23 |
| <65 | 2819 | 362 (12.8) | 0.88 (0.81~0.94) |  |
| ≧65 | 1759 | 617 (35.1) | 0.92 (0.87~0.98) |  |
| Sex |  |  |  | 0.02 |
| Male | 2344 | 564 (24.1) | 0.31 (0.18~0.54) |  |
| Female | 2234 | 415 (18.6) | 0.96 (0.9~1.02) |  |
| Residence |  |  |  | 0.79 |
| Rural | 2877 | 635 (22.1) | 0.91 (0.86~0.96) |  |
| Urban | 1701 | 344 (20.2) | 0.91 (0.83~0.99) |  |
| Marital, Status |  |  |  | 0.96 |
| Married and living with a spouse | 3916 | 719 (18.4) | 0.90 (0.86~0.95) |  |
| Married but living without a spouse | 135 | 26 (19.3) | 0.93 (0.71~1.23) |  |
| Single, divorced, and windowed | 527 | 234 (44.4) | 0.93 (0.83~1.03) |  |
| Education Status |  |  |  | 0.03 |
| Elementary school or below | 2823 | 791 (28.0) | 0.93 (0.88~0.98) |  |
| Middle school or above | 1755 | 188 (10.7) | 0.81 (0.73~0.90) |  |
| Smoking Status |  |  |  | 0.09 |
| Yes | 1212 | 572 (47.2) | 0.88 (0.82~0.94) |  |
| No | 3366 | 407 (12.1) | 0.94 (0.88~1.00) |  |
| Drinking Status |  |  |  | 0.19 |
| NO | 2451 | 299 (12.2) | 0.95 (0.88~1.02) |  |
| Yes | 2127 | 680 (32) | 0.89 (0.84~0.95) |  |
| BMI group |  |  |  | 0.74 |
| Underweight | 236 | 30 (12.7) | 0.92 (0.74~1.14) |  |
| Normal | 2683 | 430 (16) | 0.93 (0.87~0.99) |  |
| Overweight | 1347 | 370 (27.5) | 0.89 (0.82~0.96) |  |
| Obesity | 244 | 132 (54.1) | 0.89 (0.73~1.1) |  |
| Number of chronic conditions |  |  |  | 0.72 |
| 0 | 1323 | 194 (14.7) | 0.95 (0.85~1.06) |  |
| 1 | 1123 | 222 (19.8) | 0.91 (0.83~1.00) |  |
| ≧2 | 2132 | 563 (26.4) | 0.90 (0.85~0.95) |  |
| Anemia status |  |  |  | 0.77 |
| No | 3906 | 781 (20) | 0.96 (0.9~1.03) |  |
| Yes | 672 | 198 (29.5) | 0.95 (0.83~1.09) |  |
| Adjusted for age, gender, educational level, marital status, residence, smoking status, drinking status, BMI, and 14 chronic diseases. Abbreviations: OR, odds ratio; 95% CI, 95% confidence interval. | | | | |
