# Supplementary material for: Association between sleep duration and sarcopenic obesity: The mediating role of hemoglobin level
Source: PLoS One. 2026 Apr 27;21(4):e0347177. doi: 10.1371/journal.pone.0347177 (PMC13119890; doi:10.1371/journal.pone.0347177)
Supplement: S4 Table — (DOC) [file pone.0347177.s004.doc]

S4 Table. Subgroup analysis of the association between hemoglobin level with and sarcopenic obesity

| Subgroup | n.total | | n.event | | OR (95%CI) | | P for interaction |
| --- | --- | --- | --- | --- | --- | --- | --- |
| Age |  | |  | |  | | 0.26 |
| <65 | 2819 | | 362 (12.8) | | 0.72(0.66~0.79) | |  |
| ≧65 | 1759 | | 617 (35.1) | | 0.77(0.72~0.83) | |  |
| Sex |  | |  | |  | | 0.04 |
| Male | 2344 | | 564 (24.1) | | 0.94(0.83~1.00) | |  |
| Female | 2234 | | 415 (18.6) | | 0.95(0.89~1.02) | |  |
| Residence |  | |  | |  | | 0.867 |
| Rural | 2877 | | 635 (22.1) | | 0.76(0.71~0.82) | |  |
| Urban | 1701 | | 344 (20.2) | | 0.73(0.67~0.81) | |  |
| Marital, Status |  | |  | |  | | 0.473 |
| Married and living with a spouse | 3916 | | 719 (18.4) | | 0.74 (0.7~0.79) | |  |
| Married but living without a spouse | 135 | | 26 (19.3) | | 0.68(0.45~1.04) | |  |
| Single, divorced, and windowed | 527 | | 234 (44.4) | | 0.8 (0.7~0.91) | |  |
| Education Status |  | |  | |  | | 0.001 |
| Elementary school or below | 2823 | | 791 (28) | | 0.72(0.67~0.77) | |  |
| Middle school or above | 1755 | | 188 (10.7) | | 0.86(0.77~0.96) | |  |
| Smoking Status |  | |  | |  | | <0.001 |
| Yes | 1212 | | 572 (47.2) | | 0.53 (0.48~0.6) | |  |
| No | 3366 | | 407 (12.1) | | 0.92(0.86~0.99) | |  |
| Drinking Status |  | |  | |  | | <0.001 |
| NO | 2451 | | 299 (12.2) | | 0.85(0.78~0.92) | |  |
| Yes | 2127 | | 680 (32) | | 0.7 (0.65~0.76) | |  |
| BMI group |  | |  | |  | | <0.001 |
| Underweight | 236 | | 30 (12.7) | | 1.04 (0.78~1.4) | |  |
| Normal | 2683 | | 430 (16) | | 0.69(0.63~0.75) | |  |
| Overweight | 1347 | | 370 (27.5) | | 0.79(0.72~0.87) | |  |
| Obesity | 244 | | 132 (54.1) | | 0.98(0.77~1.24) | |  |
| Number of chronic conditions |  | |  | |  | | 0.345 |
| 0 | 1323 | | 194 (14.7) | | 0.73(0.64~0.82) | |  |
| 1 | 1123 | | 222 (19.8) | | 0.71 (0.63~0.8) | |  |
| ≧2 | 2132 | | 563 (26.4) | | 0.78(0.72~0.84) | |  |
| Anemia status |  |  | |  | | 0.77 | |
| No | 3906 | 781 (20) | | 0.96 (0.9~1.03) | |  | |
| Yes | 672 | 198 (29.5) | | 0.95 (0.83~1.09) | |  | |
| Adjusted for age, gender, educational level, marital status, residence, smoking status, drinking status, BMI, and 14 chronic diseases. Abbreviations: OR, odds ratio; 95% CI, 95% confidence interval. | | | | | | | |
