# Supplementary material for: Association between sleep duration and sarcopenic obesity: The mediating role of hemoglobin level
Source: PLoS One. 2026 Apr 27;21(4):e0347177. doi: 10.1371/journal.pone.0347177 (PMC13119890; doi:10.1371/journal.pone.0347177)
Supplement: S5 Table — (DOC) [file pone.0347177.s005.doc]

S5 Table. Associations of sleep duration and hemoglobin level with SO

| Variables | Model 1 | | | Model 2 (with mediator) | | |
| --- | --- | --- | --- | --- | --- | --- |
|  | Cs | SE | P value | Cs | SE | P value |
| Age | 5.73×10^−3^ | 6.76×10^−4^ | < 0.001 | 5.57×10^−3^ | 6.90×10^−4^ | < 0.001 |
| Sex (reference = Male) | -7.95×10^−1^ | 2.23×10^−2^ | < 0.001 | -7.89×10^−1^ | 2.29×10^−2^ | < 0.001 |
| Residence (reference = Urban) | -5.92×10^−3^ | 1.22×10^−2^ | 0.62 | -5.68×10^−3^ | 1.22×10^−2^ | 0.64 |
| Marital status (reference = Married and living with a spouse) |  |  |  |  |  |  |
| Married but living without a spouse | 1.21×10^−2^ | 9.60×10^−3^ | 0.20 | -3.01×10^−2^ | 3.46×10^−2^ | 0.38 |
| Single, divorced, and windowed | -9.31×10^−3^ | 3.90×10^−2^ | 0.81 | -7.03×10^−3^ | 3.91×10^−2^ | 0.85 |
| Education Status (reference = Elementary school or below) | -3.06×10^−2^ | 1.25×10^−2^ | 0.01 | -3.05×10^−2^ | 1.25×10^−2^ | 0.01 |
| Smoking Status (reference = Non-smoker) | -1.37×10^−2^ | 1.53×10^−2^ | 0.36 | -1.30×10^−2^ | 1.53×10^−2^ | 0.39 |
| Drinking Status (reference =Non-drinker) | -2.05×10^−2^ | 1.21×10^−2^ | 0.09 | -1.90×10^−2^ | 1.22×10^−3^ | 0.11 |
| BMI | 6.06×10^−4^ | 4.95×10^−4^ | 0.22 | 5.54×10^−4^ | 5.07×10^−4^ | 0.27 |
| PA (reference = Low PAL) |  |  |  |  |  |  |
| Moderate PAL | 1.70×10^−1^ | 2.85×10^−2^ | 0.15 | 1.73×10^−1^ | 2.86×10^−2^ | 0.16 |
| High PAL | 5.67×10^−2^ | 2.69×10^−2^ | 0.81 | 5.83×10^−2^ | 2.70×10^−2^ | 0.04 |
| Number of chronic conditions(reference = 0) |  |  |  |  |  |  |
| 1 | 7.78×10^−3^ | 1.44×10^−4^ | 0.59 | 7.62×10^−3^ | 1.44×10^−2^ | 0.59 |
| ≥2 | -5.52×10^−3^ | 1.62×10^−2^ | 0.73 | -5.73×10^−3^ | 1.62×10^−2^ | 0.72 |
| Abbreviations: Cs, coefficients; SE, Standard Error of the Regression Coefficient; SO, sarcopenic obesity; BMI, body mass index; PAL, physical activity level. | | | | | | |
