# Supplementary figures and images for: Association between sleep duration and sarcopenic obesity: The mediating role of hemoglobin level
Source: PLoS One. 2026 Apr 27;21(4):e0347177. doi: 10.1371/journal.pone.0347177 (PMC13119890; doi:10.1371/journal.pone.0347177)

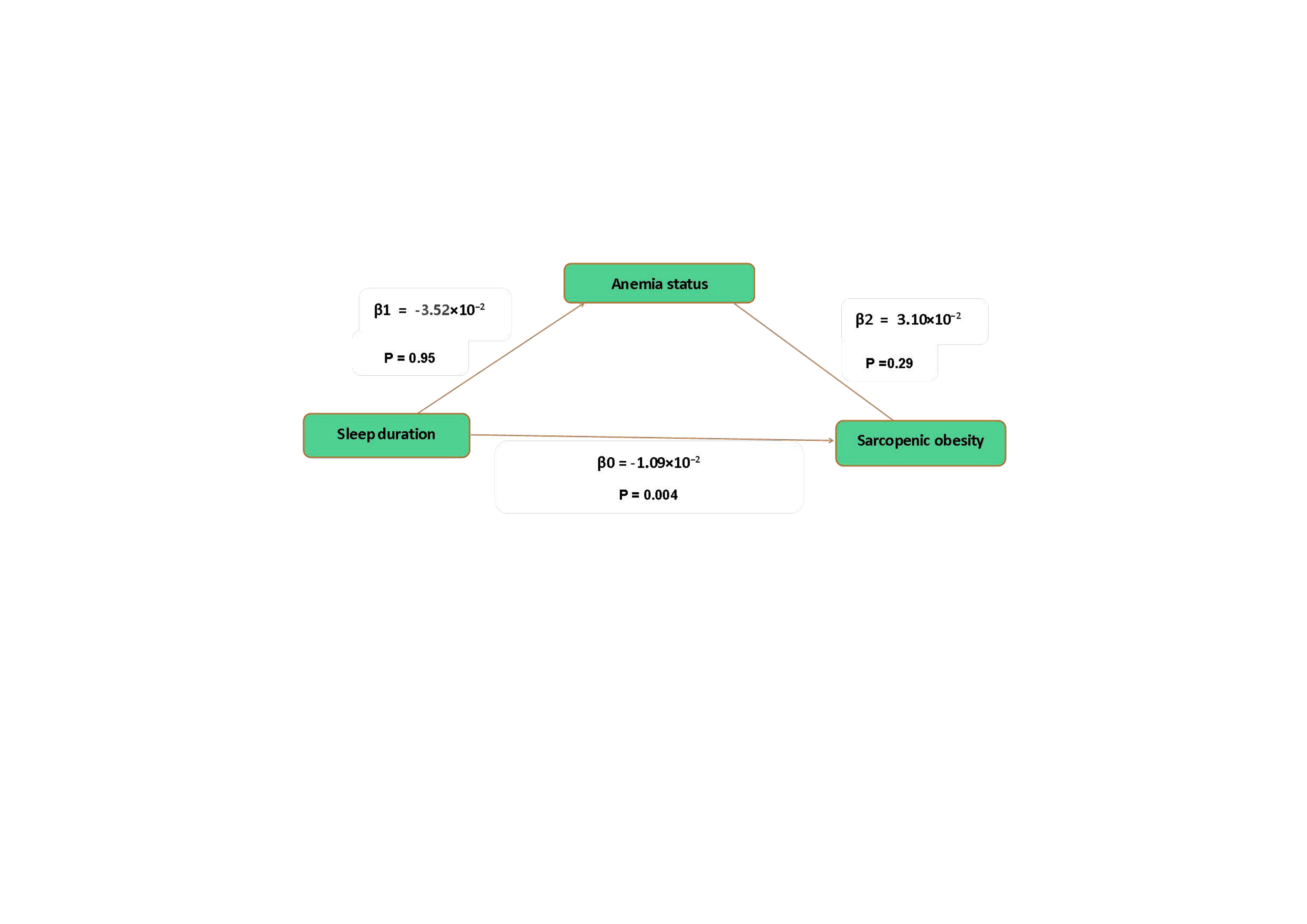

Supplement: S1 Fig — (TIFF) [file pone.0347177.s006.tiff]
